# Gene-expression of metastasized versus non-metastasized primary head and neck squamous cell carcinomas: A pathway-based analysis

**DOI:** 10.1186/1471-2407-8-168

**Published:** 2008-06-10

**Authors:** Erik F Hensen, Maria J De Herdt, Jelle J Goeman, Jan Oosting, Vincent THBM Smit, Cees J Cornelisse, Robert J Baatenburg de Jong

**Affiliations:** 1 Department of Otolaryngology and Head and Neck Surgery, Leiden University, Medical Centre, Leiden, The Netherlands; 2 Department of Pathology, Leiden University Medical Centre, Leiden, The Netherlands; 3 Department of Medical Statistics, Leiden University Medical Centre, Leiden, The Netherlands; 4 Department of Otolaryngology and Head and Neck Surgery, Erasmus Medical Centre Rotterdam, The Netherlands

## Abstract

**Background:**

Regional lymph node metastasis is an important prognostic factor in head and neck squamous cell carcinoma (HNSCC) and plays a decisive role in the choice of treatment. Here, we present an independent gene expression validation study of metastasized versus non-metastasized HNSCC.

**Methods:**

We used a dataset recently published by Roepman et al. as reference dataset and an independent gene expression dataset of 11 metastasized and 11 non-metastasized HNSCC tumors as validation dataset. Reference and validation studies were performed on different microarray platforms with different probe sets and probe content. In addition to a supervised gene-based analysis, a supervised pathway-based analysis was performed, evaluating differences in gene expression for predefined tumorigenesis- and metastasis related gene sets.

**Results:**

The gene-based analysis showed 26 significant differentially expressed genes in the reference dataset, 21 of which were present on the microarray platform used in the validation study. 7 of these genes appeared to be significantly expressed in the validation dataset, but failed to pass the correction for multiple testing. The pathway-based analysis revealed 23 significant differentially expressed gene sets, 7 of which were statistically validated. These gene sets are involved in extracellular matrix remodeling (MMPs, MMP regulating pathways and the uPA system), hypoxia and angiogenesis (HIF1α regulated angiogenic factors and HIF1α regulated invasion).

**Conclusion:**

Pathways that are differentially expressed between metastasized and non-metastasized HNSCC are involved in the processes of extracellular matrix remodeling, hypoxia and angiogenesis. A supervised pathway-based analysis enhances the understanding of the biological context of the results, the comparability of results across different microarray studies, and reduces multiple testing problems by focusing on a limited number of pathways of interest instead of analyzing the large number of probes available on the microarray.

## Background

Head and neck squamous cell carcinoma is a relatively common malignancy, associated with severe disease- and treatment-related morbidity. One of the most predictive factors of poor clinical outcome is the presence of regional lymph node metastasis, and nodal status of the neck plays a decisive role in the choice of treatment [1,2]. The complex process of metastasis in HNSCC is still incompletely understood at a molecular level; however, multiple marker studies have been performed in order to identify markers that predict the presence of metastasis. Recently, high-throughput gene expression studies have been able to identify a metastatic gene expression signature in primary HNSCC tumors, and Roepman et al. were able to predict the presence of lymph node metastasis based on gene expression of the primary tumor [3]. Analyses in this study were performed in a 'data-driven' way, by means of computational statistics without prior implementation of existing knowledge about functionally related genes and pathways. This technique is very useful in the search for new biomarkers, new subgroups, and differences in their gene expression profiles. Although the authors were able to identify a number of genes that are known to be involved in metastatic disease within this gene set, the interpretation of statistical differences in a meaningful molecular biological context is not self-evident. It has been demonstrated that classification gene sets are profoundly influenced by the microarray methodology, such as the microarray technique, microarray platform, and preprocessing methods [4-8]. Furthermore, it has been shown that classifying gene sets are highly dependent on the chosen analysis strategy [9,10]. This is illustrated by the fact that the authors were able to generate several different classifying gene sets that were all able to predict nodal metastasis with reasonable accuracy [10]. The dependence of classifying gene sets on statistical methods, as well as technical methods such as choice of microarray platform, hampers comparability of results from different microarray studies and raises questions about the biological relevance of the classifying genes. It is therefore necessary that differences in gene expression are validated in an independent dataset. Here, we present an independent gene expression validation study of metastasized versus non-metastasized HNSCC. Differences in gene expression between metastasized and non-metastasized HNSCC were determined in the publicly available dataset generated by Roepman et al., and subsequently validated in an independent gene expression dataset of 11 metastasized and 11 non-metastasized HNSCC tumors of three anatomical localizations (the oral cavity, the oropharynx and the larynx). In addition to the validation of individual differentially expressed genes, we performed a supervised, pathway-based analysis. Gene expression was evaluated within predefined subgroups of genes with a known biological context, i.e. genes within a metastasis related pathway. First, pathways and functional gene clusters that are involved in the process of metastasis in carcinoma were defined using pathways described in literature and the publicly available Kyoto Encyclopedia of Genes and Genomes (KEGG) and Biocarta pathway databases, with a focus on pathways involved in survival, proliferation, apoptosis, cell adhesion, extra cellular matrix signaling and remodeling, hypoxia and angiogenesis [11-13]. Using this supervised analysis strategy, we found considerable concordance between the datasets for pathways involved in survival, proliferation, apoptosis, cell adhesion, extra cellular matrix signaling and remodeling, hypoxia and angiogenesis. Gene sets that were validated by the independent validation dataset were matrix metalloproteinases (MMPs) and pathways involved in MMP regulation, the uPA system and pathways involved in uPA regulation, and HIF1α regulated invasion and angiogenesis. This approach to microarray analysis generates an outcome with readily interpretable biological meaning. Furthermore, by concentrating on groups of genes with a known biological relation rather than individual genes, comparability of microarray studies performed on different microarray platforms is improved [14].

## Methods

### Reference dataset

The publicly available HNSCC gene expression dataset published by Roepman and co-workers was used as the reference dataset [3,15]. All 104 samples (49 non-metastasized and 55 metastasized primary head and neck carcinoma's) analyzed in this study were included the reference dataset (Table 1). For sample selection, cRNA preparation, microarray hybridization and normalization methods in this study see elsewhere [3].

**Table 1:** Characteristics of tumors included in the reference and validation dataset

**Characteristic**	**Reference set**	**Validation set**
*Sex (n – %)*		
Male	60 (58%)	14 (64%)
Female	44 (42%)	8 (36%)
		
*Location of primary tumor (n – %)*		
oral cavity	87 (84%)	7 (32%)
oropharynx	17 (16%)	7 (32%)
larynx	0 (0%)	8 (36%)
		
*Primary tumor size (n – %)*		
*Maximal diameter (cm.)*		
< 2.5	33 (32%)	0 (0%)
2.5 – 5.0	49 (47%)	13 (59%)
> 5.0	22 (21%)	9 (41%)
		
*Nodal metastasis (n – %)*		
no (N0)	49 (47%)	11 (50%)
yes (N+)	55 (53%)	11 (50%)

### Validation dataset

#### Sample selection

An independent validation dataset was constructed using fresh frozen tumor tissue of 24 patients with an HNSCC originating from the oral cavity, the oropharynx, or the larynx. Primary tumors were removed between 1990 and 2000. Specimens were selected from the frozen tissue bank of the Leiden University Medical Centre (LUMC), the Netherlands. Neck dissection specimens of all cases were available for histological evaluation of lymph nodes. Based on histology obtained from surgical specimens as well as clinical and radiological data collected during a follow-up period of four years or more, the group was divided into a non-metastasized and a metastasized subgroup. The non-metastasized group consisted of 12 tumors (4 from the oral cavity, 4 oropharyngeal and 4 laryngeal HNSCC) from patients without indication of metastasis at the time of surgery, nor during the follow-up period. The metastasized group also consisted of 12 tumors (4 from the oral cavity, 4 oropharyngeal and 4 laryngeal HNSCC) from patients with lymph node metastasis at the time of surgery or during the follow-up period (Table 1). In all cases fresh frozen tissue of the primary tumor containing a tumor percentage of 50% or more, paraffin material, an unequivocal pathological classification of the resection material and clinical data were available. Patients who had undergone radiotherapy before surgical excision of the tumor or had a previous malignancy in the same region were excluded. All specimens were handled according to the ethical guidelines, as described in the Code for Proper Secondary Use of Human Tissue in the Netherlands of the Dutch Federation of Medical Scientific Societies (FEDERA). The data discussed in this publication have been deposited in NCBIs Gene Expression Omnibus (GEO), and are accessible through GEO Series accession number GSE9349 [16].

#### High-density oligonucleotide arrays

Data from the reference dataset were obtained with the Human Array-Ready Oligo set, version 2.0 (Qiagen) printed on Corning UltraGAPS slides, containing over 21,000 genes. These data were compared to the data from the validation set, obtained with Affymetrix HG-Focus Target arrays (Affymetrix, Inc., Santa Clara, CA) as microarray platform, containing over 190,000 unique oligonucleotides, representing more than 8,500 of the best characterized human genes.

#### Total RNA isolation, probe preparation and hybridization to arrays

Total RNA isolation, probe preparation and hybridization to arrays were performed according to the Affymetrix protocols. In short, total RNA was extracted from fresh frozen tumor sections using Trizol (Invitrogen, San Diego, CA). Total RNA was precipitated with glycogen and isopropanol, washed with ethanol and purified using the RNeasy Mini Kit (Qiagen, Valencia, CA). Total RNA yield and purity were measured using spectrophotometric analysis and in addition RNA quality was checked on a 1% agarose gel. Double-stranded cDNA synthesis was performed using the Superscript Choice system (Life Technologies, Rockville, MD); incorporating the T7 RNA polymerase promoter in the first round. The resulting ds cDNA was purified using the QIAquick PCR Purification Kit (Qiagen, Valencia, CA), and applied as a template for in vitro transcription using the RNA Transcript Labeling Kit (Enzo Diagnostics, Inc., Farmingdale, NY), incorporating biotinylated ribonucleotides required for the staining procedures after hybridization. The resulting cRNA was purified as described above and cRNA quantity and quality were measured using spectrophotometric analysis and the 2100 Bioanalyzer (Agilent, Palo Alto, CA). 15 μg fragmented, biotinylated cRNA was hybridized to the Affymetrix HG-Focus Target arrays at 45°C for 16 h. After hybridization the arrays were washed, stained and scanned, as described in the Affymetrix users' manual.

### Statistical analysis

#### Normalization and expression analysis

In the validation study, acquisition and quantification of array images was performed using the MAS software package (Affymetrix). All arrays were normalized with gcrma normalization and custom probe definitions based on EntrezGene identifiers using the R statistical software package available on Bioconductor [17-20].

#### Supervised analysis

The R package 'Linear Models for Microarray Data' (LIMMA) was used for the assessment of differential expression between N0 and N+ HNSCC subgroups in the reference dataset [21]. Multiple testing correction was performed using False Discovery Rate (FDR) analysis [22]. The reference dataset was used as the hypothesis generating dataset, and genes with a FDR adjusted p-value < 0.1 were included in the gene set that was subsequently validated using the independent validation dataset. Genes were considered to be validated by the validation dataset if an FDR adjusted p-value < 0.05 was reached.

In the study by Roepman et al. it was reported that longer sample storage time had an adverse effect on the predictive value of classifying genes, and that the classifying gene set based on samples stored after 1998 had a higher accuracy [3]. In order to evaluate whether storage time had an effect on the gene-based analysis in this study, we performed an additional LIMMA analysis on samples stored after 1998 in the reference dataset and subsequently validated the outcome using samples stored after 1998 in the validation dataset.

#### Pathway selection and analysis

In order to acquire metastatic potential, a primary tumor cell must complete a series of sequential steps, including progressive growth at the primary tumor site, vascularisation of the primary tumor, invasion of the surrounding stroma, detachment from other tumor cells, embolization of tumor cells in blood vessels or lymph vessels, survival within these vessels, extravasation and proliferation at the metastatic site [23]. Each of these steps is regulated by transient or permanent changes in DNA, RNA or proteins. We have defined pathways and gene sets involved in each of these steps using the publicly available pathway databases KEGG and Biocarta, and by researching literature on metastasis in (head and neck) carcinoma [11-13]. We categorized these pathways in the following subgroups: survival, proliferation, differentiation, apoptosis, cell adhesion, extra cellular matrix signaling and remodeling, hypoxia and angiogenesis. In all, 241 gene sets were thus created. In order to determine if gene expression was significantly different between metastasized and non-metastasized HNSCC in the reference dataset, we performed the global test designed by J.J. Goeman (available as the R package 'globaltest' at Bioconductor) on each predefined pathway [19,20,24]. The reference dataset was used as the hypothesis generating dataset, and all pathways with a FDR adjusted p-value < 0.1 were accepted for validation in the independent validation dataset. Pathways were considered to be validated by the validation dataset if an FDR adjusted p-value < 0.05 was reached.

## Results

### Unsupervised analysis of the validation dataset

2 out of 24 hybridizations were of insufficient quality, leaving 22 samples in the analysis (11 metastasized and 11 non-metastasized samples) (Table 1). No differences were observed in total RNA and cRNA yield or quality between samples with different storage time. After normalization of the validation dataset, there was a great similarity in gene-expression profiles irrespective of their N-stage or location in the oral cavity, oropharynx, or larynx. Complete linkage two-way hierarchical clustering revealed no well-defined grouping of samples according to their N-stage or location. No grouping was found according to sample storage time. We also performed this clustering analysis separately for each subgroup (according to localization or N-stage), but found no stable clusters (data not shown).

### Supervised gene-based analysis

LIMMA analysis revealed 31 significant differentially expressed oligonucleotide transcripts within the reference dataset, when corrected for multiple testing (FDR adjusted p-value < 0.1). Twenty-six of these 31 transcripts represented well-defined genes. Twenty-one of these 26 well-defined genes were also represented on the HG U95 Av2 chip used in creating the validation dataset. Seven of these 21 genes appeared to be significantly expressed in the validation dataset (raw p value < 0.05). However, all failed to pass the correction for multiple testing (FDR adjusted p-value < 0.05). These genes are lethal giant larvae protein 2 of Drosophila (LLGL2), fibroblast activation protein alpha (FAP), urokinase plasminogen activator (PLAU), laminin beta 1 (LAMB1), musculin (MSC), and collagen type V alpha 1 and 3 subunits (COL5A1 and COL5A3) (Table 2). Next, in analogy with the study by Roepman et al., we performed LIMMA analysis on the samples stored after 1998 only (66 samples in the reference dataset and 8 in the validation dataset) [3]. This revealed 352 differentially expressed well-defined genes in the reference dataset when corrected for multiple testing. However, none of these genes could be validated (FDR adjusted p-value < 0.05) in the validation dataset (data not shown).

**Table 2:** Differentially expressed genes between N0 and N+ HNSCC as validated by the independent expression dataset

**Gene**	**GenBank ID**	**Function**	**P-Value**	**P-Value (FDR adjusted)**
*LLGL2*	NM_001031803	cell division and migration	0.011	0.116
*FAP*	NM_004460	ecm remodelling	0.013	0.116
*PLAU*	NM_002658	ecm remodelling	0.017	0.116
*LAMB1*	NM_002291	basement membrane component	0.022	0.116
*MSC*	NM_005098	transcription factor	0.030	0.126
*COL5A1*	NM_000093	ecm component	0.039	0.134
*COL5A3*	NM_015719	ecm component	0.045	0.134

*COL4A1*	NM_001845	ecm component	0.077	0.201
*LAMA4*	NM_002290	basement membrane component	0.103	0.240
*CYB5R3*	NM_000398	Metabolism	0.237	0.488
*CSTA*	NM_005213	cell envelope	0.255	0.488
*PPL*	NM_002705	cell envelope	0.283	0.496
*NTHL1*	NM_002528	DNA damage repair	0.454	0.682
*TNFAIP3*	NM_006290	inhibit *TNF*-mediated apoptosis.	0.477	0.682
*TTYH1*	NM_020659	chloride anion channel	0.487	0.682
*PALM2-AKAP2*	NM_147150	Unknown	0.617	0.809
*SCRG1*	NM_007281	response to prion-associated infection	0.727	0.866
*ADAM12*	NM_021641	ecm remodelling	0.742	0.866
*FADS1*	NM_013402	Metabolism	0.869	0.888
*SOX4*	NM_003107	apoptosis and survival	0.876	0.888
*IVL*	NM_005547	cell envelope	0.888	0.888
*FN1*	NM_212482	cell adhesion and migration	-	-
*PELO*	NM_181501	cell cycle control	-	-
*FBXL19*	NM_019085	F-box protein	-	-
*DMKN*	NM_033317	Unknown	-	-
*EPPK1*	NM_031308	cell adhesion	-	-

ecm = extra cellular matrix. P-value = raw p-value of the LIMMA analysis. P-value (FDR adjusted) = p-value of the LIMMA analysis, corrected for multiple testing. P-values for genes that were not represented on the Affymetrix HG U95Av2 microarray used for the validation dataset are missing. Differentially expressed genes are considered to be validated by the validation dataset if the FDR adjusted p-value is smaller than 0.05. Although the first 7 genes (LLGL2, FAP, PLAU, LAMB1, MSC, COL5A1 and COL5A3) score a raw p-value smaller than 0.05, none of them pass the correction for multiple testing.

### Supervised pathway-based analysis

**Figure 1:**
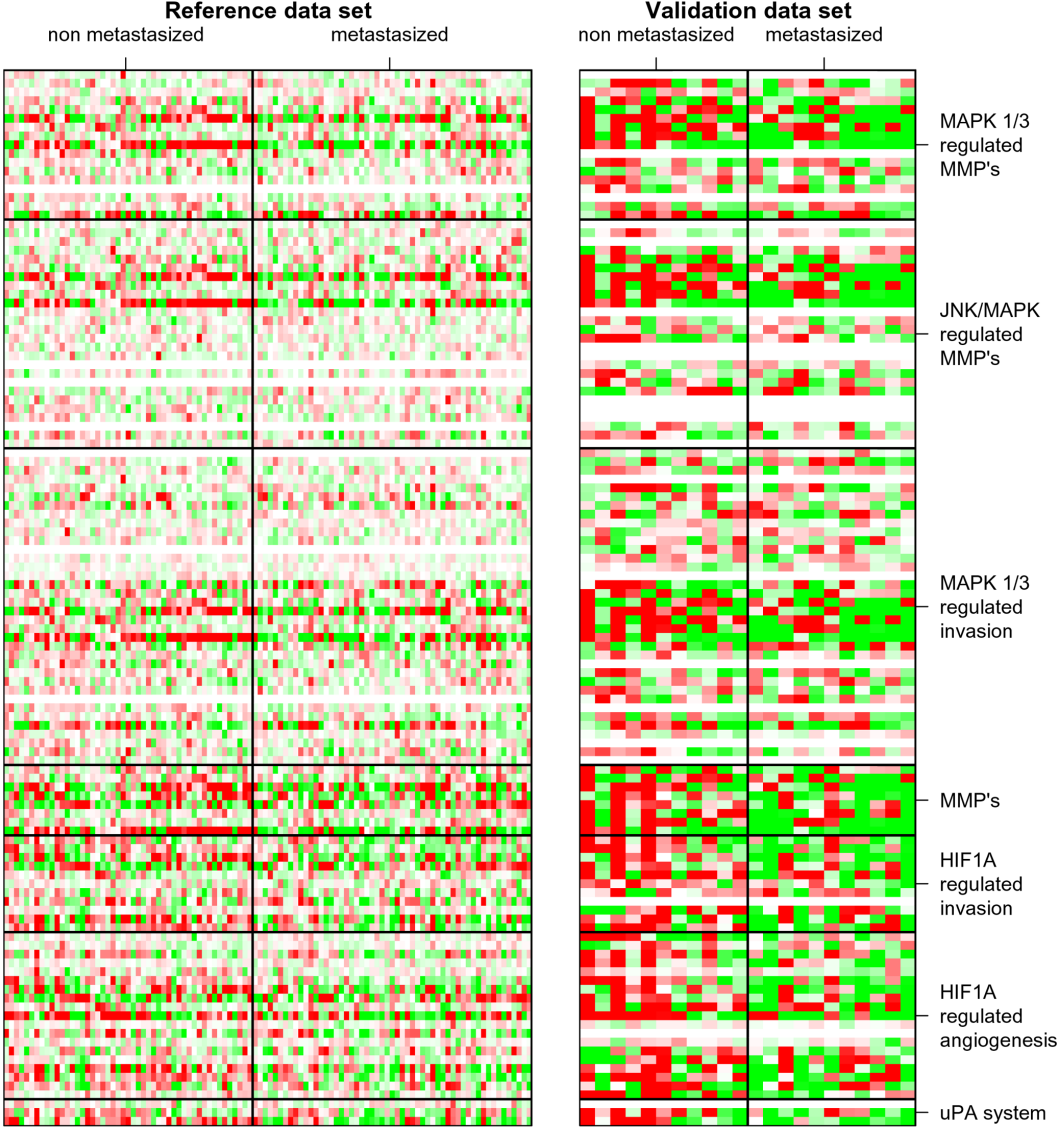
**Heatmap of pathways with validated differential gene expression between non-metastasized and metastasized primary HNSCC**. Each row in the figure denotes a gene and each column denotes a sample. Panel on the left: validated differentially expressed pathways in the reference study. Panel on the right: validated differentially expressed pathways in the validation study. Relative gene-expression is shown for each gene in the pathway, and for each sample in the N0 and N1 HNSCC subgroups, red indicating overexpression and green indicating underexpression. White brackets indicate genes not represented on the respective microarray platforms.

Analysis of the selected pathways related to survival, proliferation, apoptosis, cell adhesion, extra cellular matrix signaling and remodeling, hypoxia and angiogenesis, revealed significant differential expression for 23 gene sets in the reference dataset. These gene sets mainly encode extracellular matrix components (i.e. collagens), and pathways involved in extracellular matrix remodeling (MMPs, the uPA system, uPA regulating pathways, and HIF1α regulated invasion), hypoxia (upregulation of HIF1α target genes) and hypoxia induced angiogenesis (HIF1α regulated angiogenic factors). Of these 23 gene sets, 7 gene sets were validated by analysis of the independent expression dataset when corrected for multiple testing (Table 3 and 4). These gene sets with significant differential expression between N0 and N+ tumors in both datasets are involved in extracellular matrix remodeling (MMPs, MMP regulating pathways and the uPA system), hypoxia and angiogenesis (HIF1α regulated angiogenic factors and HIF1α regulated invasion) (Table 4 and 5, Figure 1). No significant differential expression between metastasized and non-metastasized HNSCC was shown for 215 gene sets in both datasets, including almost all survival, proliferation and apoptosis related gene sets (Table 3).

**Table 3:** Number of pathways with significant differential expression between N0 and N+ HNSCC in the reference and validation datasets

	**Pathways significant in validation dataset**	**Pathways not significant in validation dataset**
**Pathways significant in reference dataset**	7	16
**Pathways not significant in reference dataset**	3	215

In all, 241 different tumorigenesis and metastasis related pathways were tested. 23 significant differentially expressed pathways were found in the reference dataset, and 10 in the validation dataset, 7 of which overlapped. 215 pathways showed no significant differential gene expression in both datasets.

**Table 4:** Validated pathways with significant differential expression between N0 and N+ HNSCC

**Pathways**	**Genes in pathway**	**Probes in reference dataset (n)**	**Probes in validation dataset (n)**	**p-value**	**p-value (FDR adjusted)**
MAPK 1/3 regulated MMPs	16	17	14	0.003	0.028
JNK/MAPK regulated MMPs	25	28	19	0.003	0.028
MAPK 1/3 regulated invasion	35	38	30	0.004	0.028
MMPs	9	8	8	0.005	0.028
HIF1α regulated invasion	14	11	11	0.011	0.048
HIF1α regulated angiogenesis	26	21	19	0.013	0.048
uPA system	3	3	2	0.015	0.048

Genes in pathway = number of genes theoretically involved in the pathway. Probes in reference dataset = number of probes representing pathway genes in the reference dataset. Probes in validation dataset = number of probes representing pathway genes in the validation dataset. P-value = raw p-value of the global test. P-value (FDR adjusted) = p-value of the global test, corrected for multiple testing using the false discovery rate. Differentially expressed pathways are considered to be validated by the validation dataset if the FDR adjusted p-value is smaller than 0.05.

**Table 5:** Genes included in the pathways and functional gene sets that are differentially expressed between metastasized and non-metastasized HNSCC

**uPA system**	**HIF1A regulated angiogenesis**	**HIF1A regulated invasion**	**MMPs in HNSCC**	**MAPK 1/3 regulated invasion**	**JNK/MAPK regulated MMPs**	**MAPK 1/3 regulated MMPs**
PLAU	HGF	HIF1A	MMP1	SHC1	HRAS	SHC1
PLAUR	FGF2	MMP2	MMP2	GRB2	RAC1	GRB2
PLG	PDGFB	MMP13	MMP3	SOS1	PAK1	SOS1
	IL8	PLAUR	MMP6	HRAS	PAK2	HRAS
	PGF	P4HA1	MMP7	RAF1	MAP3K4	RAF1
	ANGPT2	CXCL12	MMP9	MAP2K1	MAP3K1	MAP2K1
	TEK	MMP14	MMP10	MAP2K2	MAP3K12	MAP2K2
	MMP2	COL5A1	MMP11	MAPK3	MAP2K7	MAPK3
	MMP13	CTGF	MMP14	MAPK1	MAP2K4	MAPK1
	PLAUR	ITGB2		RPS6KA5	MAPK8	FOS
	CCL2	SERPINE 1		RPS6KA1	MAPK9	MMP1
	CXCL12	CXCR 4		MKNK1	MAPK10	MMP3
	VEGFA	MET		MKNK2	JUN	MMP7
	FLT 1	ETS 1		MYC	ATF2	MMP9
	IGFBP1			ELK1	SP1	MMP10
	SERPINE 1			STAT3	ELK1	MMP13
	P4HA1			TERT	JUND	
	CXCR 4			SRF	CDC42	
	COL5A1			ELK4	CD44	
	CTGF			ATF4	MMP1	
	MMP14			FOS	MMP3	
	HIF1A			RASA2	MMP7	
	ENG			NF1	MMP9	
	EDN 1			RASA1	MMP10	
	LRP 1			RASGRF1	MMP13	
	CITED 2			RASGRP1		
				RAPGEF2		
				PRKCA		
				CD44		
				MMP1		
				MMP3		
				MMP7		
				MMP9		
				MMP10		
				MMP13		

## Discussion

It has been demonstrated that the outcome of microarray studies is profoundly influenced by the chosen analysis strategy and highly dependent on technical aspects such as sample preparation methods and choice of microarray platform [4-10]. This raises questions about the biological validity of the outcome of individual studies, and the validation of microarray studies is therefore essential. Here we present the results of an independent validation analysis of differences in gene expression between metastasized and non-metastasized HNSCC. The reference study and validation study were performed in different centers by different investigators, using different microarray platforms with different probe content. In this study, we concentrated on the validation and biological interpretation of the differences in gene expression between N0 and N+ HNSCC subgroups, and did not attempt to validate classifying gene sets that predict N-status in the reference study or other HNSCC microarray studies, because the data-driven way in which these classifying gene sets are created makes them too dependent on the microarray platform, the microarray technique, the preprocessing methods and the laboratory used to create them [4-8]. Furthermore, gaining insight into the process of metastasis on the basis of these classifying gene sets is troublesome: although some probes within the classifier encode a gene with a known role in tumorigenesis or metastasis, many others have unrelated or unknown functions [3]. The fact that multiple classifying gene sets can be constructed on basis of the reference study data casts further doubts on their biological validity [10]. In this study, a gene-based analysis revealed 7 genes that appeared to be significantly expressed in the validation dataset (raw p value < 0.05) (Table 2). All of these 7 genes are known to be involved in processes of tumorigenesis or metastasis. LLGL2 belongs to a group of genes that act as tumor suppressor genes. Loss of function is associated with disruption of cell polarity and tissue architecture, uncontrolled proliferation and growth of neoplastic lesions [25]. FAP is a cell-surface protease expressed in reactive stromal fibroblasts of epithelial cancers, and is associated with invasion and metastasis in gastric, colorectal and cervical carcinoma [26-28]. PLAU encodes a serine protease involved in degradation of the extracellular matrix. Its plays a well-known role in invasion and metastasis of carcinoma, and is a prognostic factor for metastasis and outcome [29,30]. LAMB1 encodes the β1 subunit of members from the laminin family, extracellular matrix glycoproteins that are the major non-collagenous constituent of basement membranes. Laminins containing the β1 subunit (i.e. laminin 8 and 10) have been implicated in the metastasis related processes of angiogenesis, invasion, and migration [31,32]. The protein encoded by MSC is a transcriptional repressor that attenuates E2A-mediated gene activation. MSC overexpression is associated with loss of differentiation in multiple tissues and is associated with B-cell lymphoma, but no association with epithelial cancer has been described to date [33,34]. COL5A1 and COL5A3 encode alpha chains of collagen type 5. Upregulation is associated with metastatic potential in carcinoma [3,35]. However, when corrected for multiple testing, none of these genes could be statistically validated.

Roepman et al. have reported an adverse effect of long-term storage of tissue samples on its predictive accuracy. No explanation was found for this phenomenon, but it did not seem to be attributable to differences in total RNA and cRNA yield or quality [3]. We have evaluated the effect of storage time on the gene-based validation analysis in this study. Our LIMMA analysis of the most recent tumor samples within the reference dataset identified more differentially expressed genes, an observation that seems to correlate well with the findings of Roepman et al. [3]. However, we do not find an effect of sample storage time on the outcome of our validation analysis as none of these genes are statistically validated by the most recent samples in the validation dataset.

The gene-based validation between the reference and validation studies was hampered by the use of different microarray platforms with different probes and probe content. In order to overcome this problem, a pathway-based supervised analysis was performed, evaluating differences in gene expression between metastasized and non-metastasized HNSCC for predefined tumorigenesis- and metastasis related pathways and gene sets. By analyzing groups of functionally related genes, we were able to study the same biological processes in both reference and validation datasets, even though not all genes involved in these processes were present, and the number and nature of the represented genes varied in the respective datasets. In this way, 7 metastasis-related pathways and functionally related gene sets that differentiate between metastasized and non-metastasized HNSCC were statistically validated (Table 4 and Figure 1). These validated pathways are metalloproteinases and regulatory pathways of metalloproteinases, HIF1α induced invasion- and angiogenesis related target genes and the urokinase plasminogen activator system, key pathways involved in invasion, extra cellular matrix remodelling, detachment and angiogenesis, essential steps in the progression to metastatic disease (Tables 4 and 5). Metalloproteinases play a complex role in tumor progression and metastasis. Not only do they facilitate invasion by degrading components of the extracellular matrix, there is also evidence that they are involved in angiogenesis. MMPs that induce metastasis are not only produced by the tumor cells but also by stromal cells and leucocytes, especially along the invasive front of the tumor [36]. The second messenger signalling pathways that lead to expression of MMPs are not fully understood, but there is evidence that MAPK pathways are involved [37]. Three different regulatory MAPK pathways of MMPs have been identified, and in this study two of them show significant differential expression between metastasized and non-metastasized HNSCC: the MAPK1/3 (ERK1/2) and JNK/MAPK pathways. The MAPK1/3 pathway, which is activated by a variety of mitogenic and growth factors, induces FOS and JUN phosphorylation and expression. The JNK/MAPK pathway, which is induced by various inflammatory cytokines, increases transcriptional activity and protein stability of JUN. FOS and JUN are leucine zipper proteins that can dimerize forming the AP-1 transcription factor complexes. JUN, FOS and AP-1 complexes seem to regulate expression of multiple MMPs [37]. The urokinase plasminogen activator system (uPA) mediates invasion and metastasis by catalysing extracellular matrix dissolution, and there is evidence that the uPA system plays a role in cell proliferation, migration and modulation of cell adhesion as well. The potential of components of this system as prognosticators in cancer has been evaluated most extensively in breast cancer, but also in HNSCC [38]. PLAU in particular seems to correlate well with unfavourable outcome, and in this study PLAU correlated well with metastatic HNSCC in both the reference and validation datasets. Hypoxia is a common feature in solid tumors and their metastasis, and can lead to tumor progression in a variety of ways. It induces HIF1α, a transcription factor that regulates angiogenesis as well as cell survival, invasion and metastasis by activating transcription of a host of target genes. The gene sets comprised of HIF1α target genes that are known to be involved in angiogenesis and invasion are significantly upregulated in metastasized HNSCC in this study [39-41]. As oligonucleotide microarrays measure mRNA levels, results reflect the gene-expression levels in N0 and N+ HNSCC. Post-transcriptional events such as splicing, translation or activation of the proteins encoded by these genes are not measured. Upregulation of genes in a specific pathway as determined by oligonucleotide microarrays therefore may not necessarily mean heightened activity of the pathway. However, it is very plausible that the observed differences in gene-expression levels of genes involved in metastasis-related pathways are responsible for the differences in metastatic potential of N0 and N+ HNSCC.

The aim of this study is not only to identify and validate a gene-expression profile that characterizes metastatic disease in head and neck squamous cell carcinoma, but to provide an analysis strategy that incorporates the available insights in the pathways that lead to metastasis. This supervised pathway-based analysis will not reveal new, previously unknown metastasis related biomarkers. It does however increase our understanding of the biological context of the results. By focusing on pathways and functional gene sets, rather than individual genes, the insight into the biological steps that lead from carcinogenesis to metastatic disease in HNSCC is enhanced. Furthermore, by leaving probes that are not relevant to the biological processes of interest out of the analysis, statistical noise and multiple testing problems associated with microarray analysis are reduced. The most important advantage of this strategy however is the increased comparability of data from different microarray studies. Microarray analyses based on individual genes are highly dependent on the exact gene content of the microarray used in the study, and thus on the chosen microarray platform. In a pathway-based analysis however, gene expression does not have to be measured from every single gene involved in a specific pathway, as long as a representative subset of genes is assessed. These representative subsets of genes involved in a specific pathway may vary between studies. A pathway-based analysis thus can reveal biologically relevant similarity between results of different microarray studies even though the gene contents of the microarray platforms used do not match exactly.

## Conclusion

In this gene-expression study, we were able to identify and validate several pathways that are differentially expressed between metastasized and non-metastasized HNSCC. These pathways are involved in the processes of extracellular matrix remodeling (*MMPs*, *MMP *regulating pathways and the *uPA *system), hypoxia and angiogenesis (*HIF1α *regulated angiogenic factors and *HIF1α *regulated invasion). By focusing on pathways and functional gene sets instead of individual probes in the analysis of microarray data, the biological context of the results is readily interpretable. Furthermore, a supervised, pathway-based analysis reduces multiple testing problems associated with microarray analysis by focusing on a limited number of pathways instead of analyzing all of the probes available on the microarray. Most importantly, the comparability of results from different microarray studies is greatly improved. A supervised, pathway based analysis can reveal biologically relevant similarity between results of different gene-expression studies, even if studies have used different microarray platforms with different probes and probe content.

## Abbreviations

LLGL2: lethal giant larvae homolog 2; FAP: fibroblast activation protein alpha; PLAU: plasminogen activator urokinase; LAMB1: laminin beta 1; MSC: musculin; COL5A1: collagen type V alpha 1; COL5A3: collagen type V alpha 3; MMP: matrix metalloproteinase; uPA: urokinase plasminogen activator system; HIF1α: hypoxia-inducible factor 1; MAPK: mitogen-activated protein kinase; JNK: c-jun N-terminal kinase; FOS: v-fos FBJ murine osteosarcoma viral oncogene homolog; JUN: c-jun oncogene; AP-1: DNA binding/transcription factor AP1.

## Competing interests

The authors declare that they have no competing interests.

## Authors' contributions

EFH participated in the design of the study, in the validation microarray experiments and the statistical analysis, and drafted the manuscript. MJDH carried out the validation microarray experiments and helped with the statistical analysis. JJG participated in the design of the study, carried out part of the statistical analysis and helped to draft the manuscript. JO carried out part of the statistical analysis and helped to draft the manuscript. VTHBMS helped with designing the study and drafting the manuscript. CJC participated in the design of the study, its coordination and drafting of the manuscript. RJBdJ conceived of the study, participated in its coordination and helped to draft the manuscript. All authors read and approved the final manuscript.

## Pre-publication history

The pre-publication history for this paper can be accessed here:


